# Toxoplasmosis chorioretinitis mimicking cytomegalovirus retinitis in an immunocompromised pediatric patient following bone marrow transplantation

**DOI:** 10.3205/oc000201

**Published:** 2022-06-07

**Authors:** Anubhav Garg, Bryon R. McKay, Carolina L. M. Francisconi, Rajeev H. Muni

**Affiliations:** 1 Faculty of Medicine, University of Toronto, Ontario, Canada; 2 Department of Ophthalmology and Vision Sciences, The Hospital for Sick Children, University of Toronto, Ontario, Canada; 3 Department of Ophthalmology and Vision Sciences, St. Michael’s Hospital, University of Toronto, Ontario, Canada

**Keywords:** toxoplasmosis, chorioretinitis, cytomegalovirus, immunocompromised, bone marrow transplant, pediatric

## Abstract

**Objective:**

To review a case of toxoplasmosis chorioretinitis mimicking cytomegalovirus retinitis in an immunocompromised patient following bone marrow transplantation.

**Methods:**

Retrospective chart review of a 14-year-old female who had a history of leukemia and allogeneic bone marrow transplants prior to her ocular symptoms.

**Results:**

Anterior chamber fluid analysis was positive for *Toxoplasma gondii*. The patient responded well when cytomegalovirus retinitis treatment was switched to intravitreal clindamycin with systemic sulfadiazine and prednisone.

**Conclusions:**

This case demonstrates the challenges of diagnosing and treating retinal infections in immunocompromised patients as they may present with atypical findings that mimic other pathologies and may have contraindications against standard treatment.

## Introduction

The obligate intracellular parasite *Toxoplasma gondii* can cause a devastating ocular infection known as ocular toxoplasmosis. The definitive host for *T. gondii* is the domestic cat and its relatives (Felidae). Humans become intermediate hosts through consuming contaminated undercooked meat or wild game, contaminated drinking water, close contact with infected cats, transplacentally, and, rarely, via blood transfusion or organ transplantation [1]. Ocular toxoplasmosis is typically diagnosed based on clinical findings including focal retinochoroiditis, a nearby retinochoroidal scar, and moderate to severe vitreous inflammation (vitritis) – the so-called “headlight in the fog” appearance of the fundus. However, there are many atypical retinal presentations. Some presentations mimic other pathologies, which makes it especially challenging to clinically diagnose toxoplasmosis chorioretinitis. Atypical toxoplasmosis infections can present as neuroretinitis, punctate outer retinitis (PORT), and, rarely, multifocal diffuse necrotizing retinitis that mimics viral retinitis [2], [3]. The diagnostic challenge is exacerbated in immunocompromised patients, as they may present with systemic co-infections which limit the utility of serological testing. This unusual case demonstrates toxoplasmosis chorioretinitis mimicking CMV retinitis, which is diagnosed via clinical findings including yellow-white retinal lesions that often start in the periphery, and retinal hemorrhages with a whitish, granular appearance to the retina [4].

## Case description

**Figure 1:**
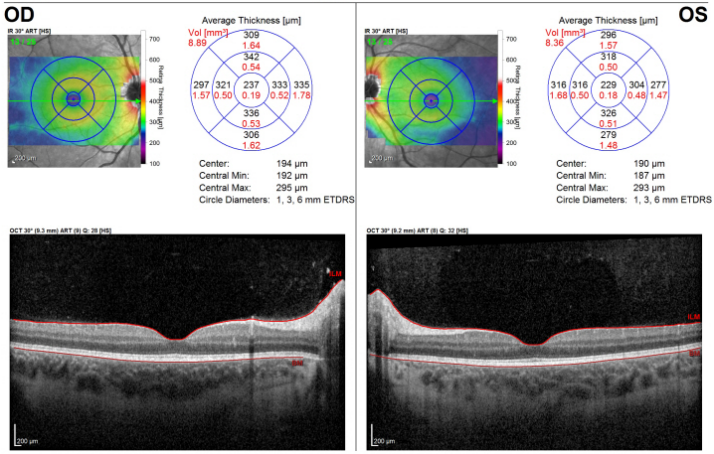
Initial presentation. Optical coherence tomography (OCT) of the right (OD) and left (OS) macula, demonstrating a normal retinal architecture of the macula and fovea in both eyes.

**Figure 2:**
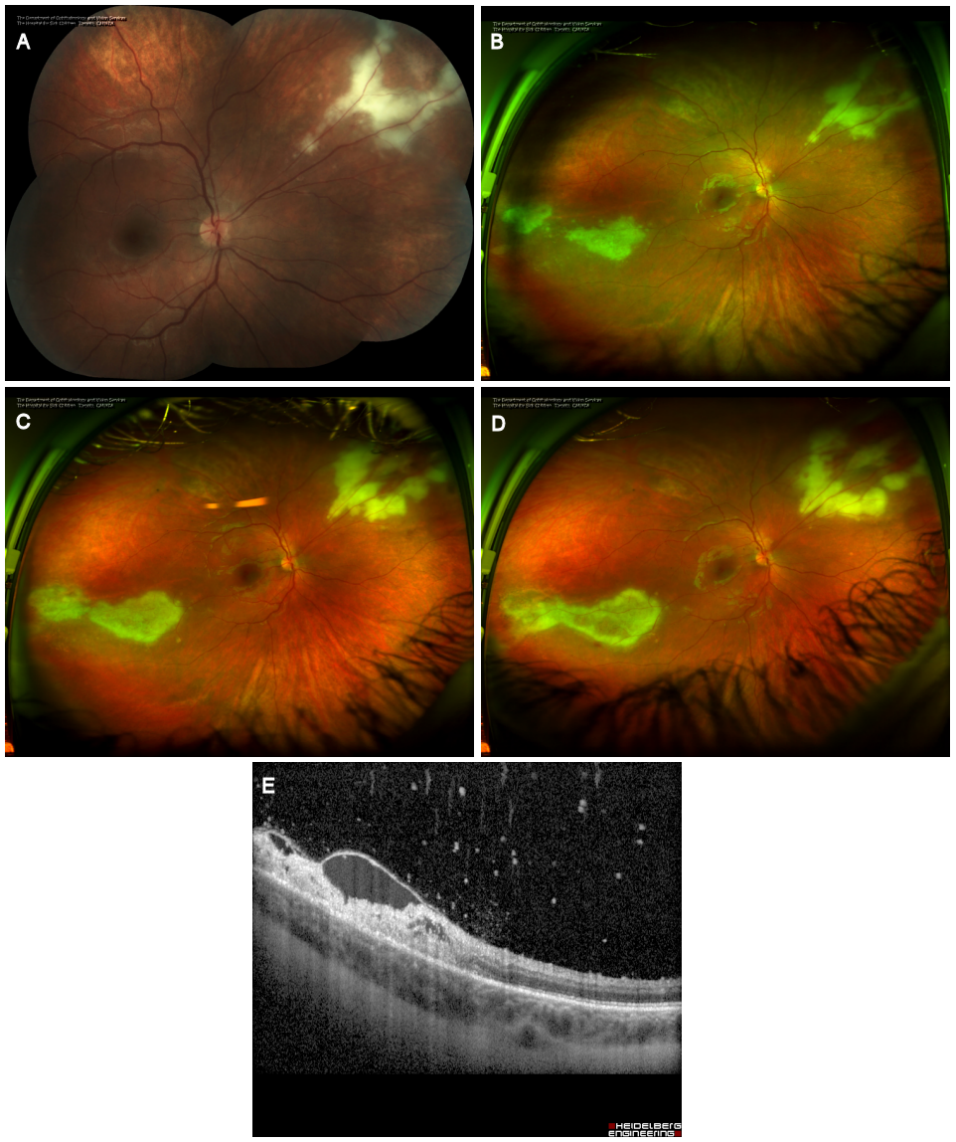
Initial presentation. (A) Color fundus montage photography of the posterior retina of the right eye. Note the white granular retinal lesion. (B) Optos ultra-widefield photography of the right fundus one weeks after the initial presentation demonstrating both supranasal and infratemporal areas of retinitis. (C) Ultra-widefield photography of the right fundus two weeks after the initial presentation demonstrating progression of the lesions despite systemic antivirals. (D) Further progression three weeks after initial presentation. (E) OCT over the edge of the infratemporal area of retinitis demonstrating focal vitritis, intraretinal fluid and retinal necrosis.

This case report has been approved by an institutional review board and the patient granted informed consent. An immunocompromised 14-year-old female with a history of acute lymphoid leukemia (ALL) and two allogeneic bone marrow transplants (BMT) in response to ALL relapses was referred to the Pediatric Ophthalmology service for blurry vision in the right eye. She was on an ITP-treosulfan-fludarabine-thiotepa-ATG conditioning regimen for the second BMT. She was also given methotrexate for graft versus host disease (GvHD) prophylaxis. She had enteric GvHD as a complication of both transplants, but both cases had resolved. On exam, she was 20/20 in both eyes with normal intraocular pressure and normal pupillary responses. Optical coherence tomography (OCT) demonstrated a normal macula. Dilated fundus examination revealed a small inferotemporal diffuse granular white deep retinal lesion (Figure 1 (Fig. 1), Figure 2 (Fig. 2)) and a superonasal retinal white lesion along the vascular arcade (Figure 1 (Fig. 1)) that was clinically consistent with viral retinitis in the context of a CMV-positive blood sample and the patient’s immunocompromised state. Treatment with oral valganciclovir was initiated and the CMV load became undetectable. However, over the next three weeks, the patient presented with progression of these retinal lesions with associated hemorrhages in the inferotemporal and superonasal quadrants in the peripheral retina of the right eye (Figure 2A (Fig. 2) (week 1), Figure 2B (Fig. 2) (week 2)). CMV retinitis was still considered as the most likely diagnosis at this time and treatment with valganciclovir was continued, rather than IV ganciclovir due to neutropenia.

**Figure 3:**
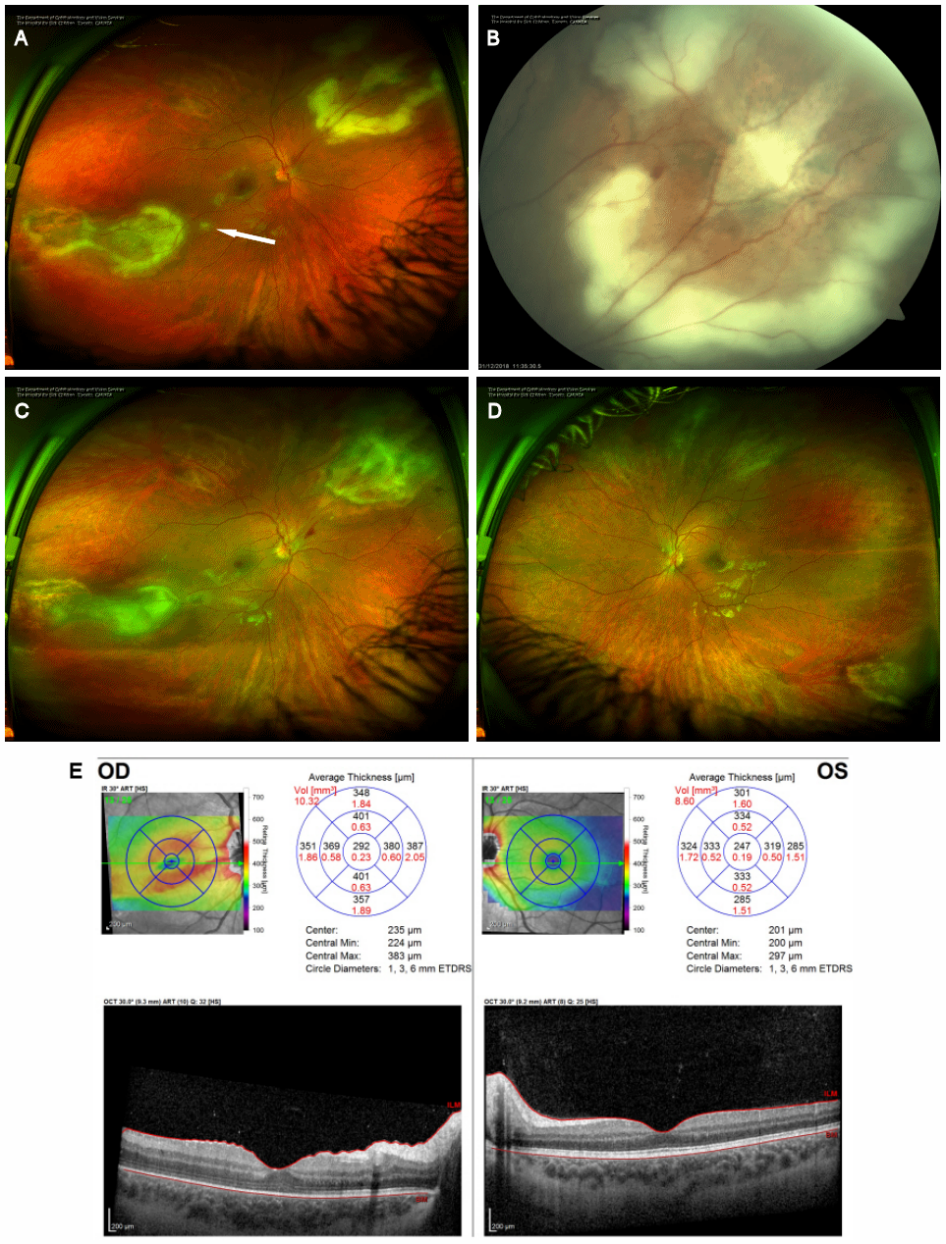
Progression despite treatment. (A) Optos ultra-widefield photography of the right fundus four weeks after the initial presentation demonstrating both supranasal and infratemporal areas of retinitis with a magnified view of the superonasal lesion demonstrating a progression of the retinitis (B). The arrow in (A) denotes a new satellite area of retinitis. (C) Optos ultra-widefield photography of the right eye and (D) left eye demonstrating the early response to the first round of intra-vitreal clindamycin and foscarnet with the lesion margins beginning to show healing. (E) OCT analysis of the macula demonstrating thickening of the nasal macula in the right eye with distortion of the architecture of the retina. The left eye remains normal.

Two weeks later, fundus exam of the right eye demonstrated a further interval progression of the patient’s retinal lesions with subtle focal vitritis and hemorrhage associated with the superonasal retinitis (Figure 2B–C (Fig. 2)). There was a new peripheral inferotemporal retinochoroidal lesion in the left eye (Figure 3A–B (Fig. 3)). The patient’s visual acuity was stable. After consultation with the Infectious Diseases service, she was switched to IV ganciclovir (2.5 mg/kg BID).

The patient presented with further progression of the retinal lesions over the next week and was referred to the Retina service for re-assessment and consideration of intravitreal antiviral therapy. Her BCVA was 20/40 in the right eye and 20/25 in the left eye. The patient’s fundus exam showed inferotemporal and superonasal white retinal lesions in the right eye, with an interval progression of the retinal lesions. The left eye showed a small inferotemporal white lesion in the far periphery, which also demonstrated interval progression.

The decision was made to inject intravitreal ganciclovir (1 mg/0.1 cc) and perform an anterior chamber paracentesis to perform viral PCR. On a subsequent visit, the patient had an intravitreal foscarnet injection (2.4 mg/0.1 cc) for possible ganciclovir-resistant CMV and clindamycin (1 mg/0.1 mL) for possible toxoplasmosis, which was considered because the patient had previously tested positive for toxoplasmosis IgG. In the same visit, anterior chamber fluid was sent for toxoplasmosis PCR.

AC fluid PCR was negative for viral etiologies including CMV. The toxoplasmosis PCR was positive, confirming the diagnosis of toxoplasmosis chorioretinitis in both eyes. Systemic therapy was required due to the ongoing immunosuppression. Furthermore, an MRI of the brain was performed to rule out cerebral disease. MRI revealed a contrast-enhancing T2 FLAIR hyperintense white matter lesion within the left posterior temporal lobe, which was suggestive of toxoplasmosis. Consequently, the patient was treated systemically with oral prednisone pulse with taper and sulfadiazine 1,500 mg twice daily. Pyrimethamine was not used due to its suppressive effects on bone marrow. IV ganciclovir was discontinued.

**Figure 4:**
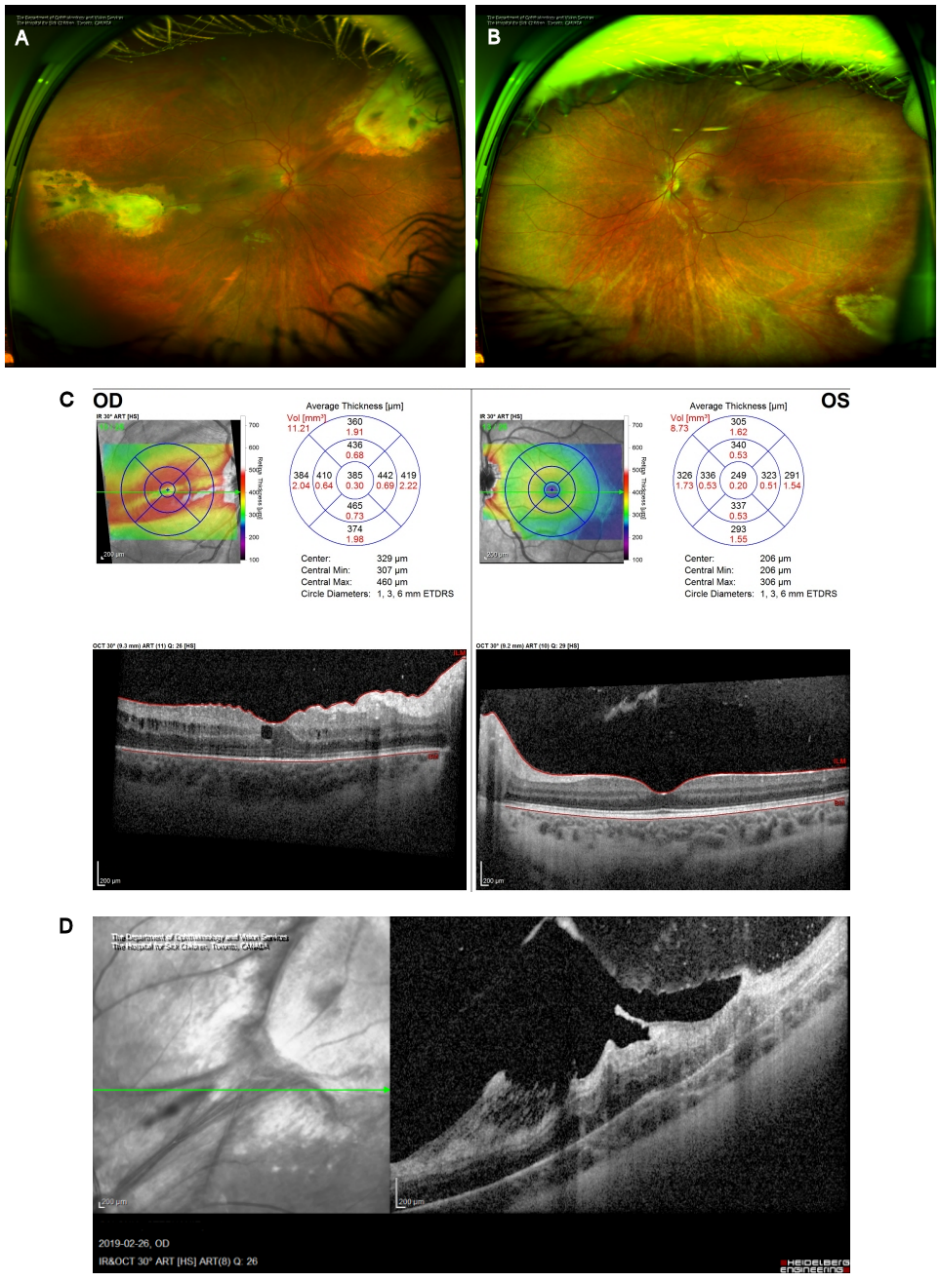
Resolution and scarring. (A) Optos ultra-widefield photography of the right eye and (B) left eye demonstrating the residual retinochoroidal scars in both eyes following successful treatment of toxoplasmosis. (C) OCT analysis demonstrating stable ERM with retail wrinkling and mild CME in the right eye and normal macula of the left eye. (D) OCT analysis of the right eye superonasal scar demonstrates a significant ERM with thickening and scarring. There is also significant retinochoridal atrophy with overlying fibrosis of the vitreous.

Six weeks later, BCVA was 20/50 +2 in the patient’s right eye and 20/20 in the left eye with correction. There was less vitritis in the right eye compared to previous exams. On fundus exam, the macula of the right eye showed mild epiretinal membranes (ERM) with mild cystoid macular edema (CME). Peripheral examination of both eyes revealed quiescent lesions (Figure 4 (Fig. 4)).

Two months later, visual acuity returned to 20/25 in the right eye and 20/20 in the left with minimal metamorphopsia in the right eye. The retinochoroidal lesions in both eyes were quiescent with stable scars (Figure 4A–B (Fig. 4)). Therapy for toxoplasmosis was discontinued.

## Discussion

This unusual case exemplifies one presentation of ocular toxoplasmosis that mimics CMV retinitis. Ocular toxoplasmosis typically presents with findings such as moderate to severe vitreous inflammation, focal retinochoroiditis, and a nearby retinochoroidal scar [2], [3]. However, in this case, the presentation was white-yellowish necrotizing retinal lesions with associated hemorrhages in the periphery, which is more typical of CMV retinitis [4]. Since CMV retinitis is largely a clinical diagnosis based on classic retinal findings and ocular toxoplasmosis is less common, an anterior chamber fluid analysis for toxoplasmosis was not initially performed. Consequently, the initial treatment was directed at CMV, and the alternate diagnosis was not made until the patient failed to improve despite treatment. The possibility of CMV ganciclovir resistance is also a significant consideration in such cases, as CMV drug resistance has been reported to be as high as 14.5% in bone marrow transplant recipients receiving prophylactic therapy [5]. The decision was made to proceed with foscarnet to cover for this possibility.

Since the diagnoses of ocular CMV and ocular toxoplasmosis are mainly clinical, aqueous sampling is mostly reserved for refractory cases and cases where the presentation is not classic (i.e., punctate outer retinal toxoplasmosis (PORT) or ill-defined retinochoroiditis, such as the present case). Classically, anterior chamber paracentesis for toxoplasmosis PCR has been difficult due to low sample volumes, 53% sensitivity, and 83% specificity of traditional PCR techniques that used the B1 gene segment for detection [6]. More recently, labs have added a dual-target assay for the B1 and Rep529 gene sequences, which enhances the sensitivity and specificity of the test to 97% and 99%, respectively [7]. This demonstrates the value in performing diagnostic anterior chamber paracentesis in such complex cases.

This case demonstrates the challenge of treating immunocompromised patients, as standard treatment may be contraindicated, necessitating alternative treatment regimens. Specifically, the patient was immunocompromised due to a history of ALL requiring BMT and thus therapies that cause bone marrow suppression are contraindicated. After she was diagnosed with toxoplasmosis chorioretinitis, a brain MRI showed white matter lesions suggestive of a possible toxoplasmosis in the left posterior temporal lobe. Thus, the patient required aggressive systemic treatment that typically involves triple therapy of systemic pyrimethamine, sulfadiazine, and corticosteroids. However, due to the toxicity of pyrimethamine on bone marrow, it was not used [8], [9]. Consequently, the patient was treated with oral prednisone and sulfadiazine.

## Conclusion

This case demonstrates the challenge of diagnosing immunocompromised patients with retinal infections, as they may present with atypical retinal findings that mimic other pathologies posing a diagnostic dilemma. This case underpins the need for prompt ophthalmologic assessment for any immunocompromised patient with visual complaints, as the first presentation of systemic infection can present with ocular findings.

## Notes

### Competing interests

The authors declare that they have no competing interests.
